# Understanding the glioblastoma tumor microenvironment: leveraging the extracellular matrix to increase immunotherapy efficacy

**DOI:** 10.3389/fimmu.2024.1336476

**Published:** 2024-02-06

**Authors:** Jimena Collado, Lauren Boland, Jared T. Ahrendsen, Jason Miska, Catalina Lee-Chang

**Affiliations:** ^1^ Department of Neurological Surgery, Feinberg School of Medicine, Northwestern University, Chicago, IL, United States; ^2^ Department of Pediatrics, Ann and Robert H. Lurie Children’s Hospital of Chicago, Chicago, IL, United States; ^3^ Department of Pathology, Feinberg School of Medicine, Northwestern University, Chicago, IL, United States; ^4^ Lurie Cancer Center, Lou and Jean Malnati Brain Tumor Institute, Chicago, IL, United States

**Keywords:** glioblastoma, extracellular matrix, tumor microenvironment, immunotherapy, cancer-associated fibroblasts, fibronectin, decorin, collagen

## Abstract

Glioblastoma (GBM) accounts for approximately half of all malignant brain tumors, and it remains lethal with a five-year survival of less than 10%. Despite the immense advancements in the field, it has managed to evade even the most promising therapeutics: immunotherapies. The main reason is the highly spatiotemporally heterogeneous and immunosuppressive GBM tumor microenvironment (TME). Accounting for this complex interplay of TME-driven immunosuppression is key to developing effective therapeutics. This review will explore the immunomodulatory role of the extracellular matrix (ECM) by establishing its contribution to the TME as a key mediator of immune responses in GBM. This relationship will help us elucidate therapeutic targets that can be leveraged to develop and deliver more effective immunotherapies.

## Introduction

Glioblastoma (GBM) accounts for approximately half of all malignant central nervous system tumors. It is one of the most aggressive malignancies, having a median overall survival of only 15 months and a five-year survival of less than 10% (1). Despite the immense advancements in the field of oncology, the standard of care treatment has not improved significantly in several decades (2, 3). The median progression-free survival after treatment is little more than 6 months (4).

Immunotherapy leverages the body’s own immune responses to specifically target and eradicate tumors. They generally fall within five categories: cancer vaccines, cytokine therapies, adoptive cell transfer, immune checkpoint inhibitors, and oncolytic therapies. Many of these therapies are becoming the additional pillar of cancer treatment by supplementing surgery, radiotherapy, chemotherapy, and targeted therapy (5). However, GBM has not benefitted from these advancements; immunotherapies such as dendritic cell vaccines, chimeric antigen receptor T cell therapy, and several checkpoint inhibitors have not delivered the expected outcomes, as they fail to show a clear survival benefit in clinical trials with high inter-patient variability (6–10). The failure of these therapies is attributed to the highly spatiotemporally heterogeneous and immunosuppressive TME (11). The TME comprises cellular and non-cellular components such as neurons, glial cells, immune cells, and extracellular matrix components (ECM). Of these components, the ECM of GBM and its interaction with immune cells is still clouded in mystery.

Around 20% of the adult brain volume is attributed to the ECM (12). The ECM of the central nervous system is unique from the rest of the body as it is predominantly non-fibrous and contains specialized forms of ECM termed perineuronal nets, as well as the meningeal and vascular basement membranes (13). The perineuronal nets vary in composition with age and location within different brain areas but are characterized by condensed regions of glycosaminoglycan-rich structures surrounding fast-spiking neurons (14). The vascular basement membrane comprises laminins, collagen IV, fibronectin, and proteoglycans essential to maintaining the blood-brain barrier (15). The brain parenchyma is composed of glycosaminoglycans like hyaluronic acid, proteoglycans, glycoproteins, and low amounts of fibrous proteins such as laminins and collagens which are synthesized by neurons and glial cells (16).

The GBM extracellular matrix is characterized by an increased overall density and tension with aberrant expression of ECM components such as hyaluronic acid (HA), tenascin-C, fibronectin, proteoglycans, and matricellular proteins (17, 18). The higher expression of extracellular molecules like tenascin-c and fibronectin predict poor prognosis in the GBM (19–21). A recent correlative study identified that there is a specific collagen signature (COL1A1, COL1A2, COL3A1, COL4A1, COL4A2, and COL5A2) that is associated with poor overall survival and increased immunosuppression (22).

## The role of ECM in tumor immunity

Besides their role as structural components, the ECM also exert profound immunomodulatory effects in solid tumors. While this topic is only recently being studied in GBM, there is a wealth of studies in other tumor types that may inform us of its role in GBM. The role of ECM in immunosuppression has been extensively explored in pancreatic ductal adenocarcinoma (PDAC) tumors. The dense and fibrotic ECM in pancreatic tumors is characterized by an abundance of collagens, fibronectin, and hyaluronic acid which create physical barriers that hinder immune cell infiltration into the tumor microenvironment (23). It is thought that this barrier limits the access of cytotoxic T cells and other immune effector cells, reducing their ability to recognize and attack cancer cells.

Another mechanism through which the ECM can hinder or potentiate immunotherapies is its role in leukocyte localization and infiltration. Leukocytes must arrive at the tumor through vasculature and then overcome the blood-brain barrier (24). It is of note that CAFs release vascular endothelial growth factor (VEGF) to target the endothelial membrane and, through aberrant angiogenesis, can mediate the localization of leukocytes in the tumor vasculature (25). Since aberrant neovascularization is another characteristic of the heterogeneous TME that promotes tumorigenesis (26), its modulation by ECM components further emphasizes their importance.

To infiltrate the tumor, leukocytes must cross the endothelial basement membrane. The biochemical composition of these membranes can determine the sites of T cells and monocyte extravasation: laminin α4 defines the sites apt for leukocyte extravasation, in opposition to laminin α5, which inhibits T cell, neutrophil, and monocyte transmigration (27). Afterward, the transmigration across the parenchymal basement membrane is mediated by macrophage-released MMP2 and MMP9 (28). Overall, lymphocytic infiltration occurs due to abnormal vessel formation and decreased adhesion molecules (29). ECM components can aid or hinder anti-tumor immunity by mediating leukocyte infiltration. Regarding the specific components of ECM, below we will discuss their known roles in immunity and GBM, starting with the major producer of ECM, fibroblasts.

### CAFs and collagen

Fibroblasts are the cell population responsible for the production of ECM components, particularly collagen (30). As they have historically been found within fibrous tumors such as lung adenocarcinoma and pancreatic ductal adenocarcinoma (PDAC), they have been given the title of cancer-associated fibroblasts (CAFs) (31, 32). In pancreatic cancer, excessive deposition of collagen by CAFs is thought to inhibit CD8+ T cell infiltration into the tumor (33). It was found that PDAC produce unique Collagen 1 (Col1) homotrimers, promoting oncogenic signaling, tumor growth, and immunosuppression, and their deletion enhances overall survival, T cell infiltration, and response to anti-PD-1 immunotherapy, suggesting a potential cancer-specific therapeutic target (34). In lung tumors, those resistant to PD-1/PD-L1 blockade therapy had an increase in collagen levels and a decreased total CD8+ T cell population. This study also found that the blockade of the immune-inhibitory transmembrane receptor LAIR-1, with a high affinity for collagen, sensitized tumors resistant to anti-PD-1 therapy and decreased CD8+ T cell exhaustion (35). These results can potentially be extended to GBM, as Xu et al., also found LAIR-1 expression to be upregulated in GBM (36).

However, this is conflicting evidence to these observations: A seminal study in PDAC tumors identified that depletion of CAFs from pancreatic tumors instead increased malignancy, and promoted immunosuppression, through the increase of regulatory T-cell populations (37). Indeed, a follow-up study specifically depleted collagen production in myofibroblasts which enhanced tumor growth and promoted immunosuppression in murine models of PDAC (38). Therefore, the role of cancer-associated fibroblasts (CAFs) in immunosurveillance remains controversial.

Despite the somewhat conflicting roles of CAFs in immunosuppression in these tumors, there are recent studies indicating that manipulating the ECM can promote immunotherapeutic efficacy. A recent study was able to use collagen overexpression in tumor as a way to increase immunotherapy efficacy (34). In this study, the author’s linked immune checkpoint inhibitor antibodies (anti-CTLA4 and anti- PD-L1) to a collagen-binding domain (CBD) derived from the blood protein von Willebrand factor (VWF) A3 domain. This leveraged the tumor vasculature’s leakiness, exposing the tumor stroma collagen to blood components. The therapy decreased the systemic toxicities observed with standard therapy, increased CD8+ T cell infiltration, and had significant anti-tumoral activity in murine models for skin, colon, and breast cancer.

Recent findings are beginning to uncover the importance of CAFs and the ECM in GBM. CAFs were identified in glioblastoma through spatial transcriptomics and single-cell RNA sequencing experiments that revealed pro-tumoral effects through their interaction with glioblastoma stem cells (39). This same study observed the recruitment of CAFs to the perivascular niche in GBM by glioma stem cells. CAFs also promoted GBM cell survival by conferring them resistance to ferroptosis by upregulating the long noncoding RNA DLEU1 (40). Importantly, CAFs are major producers of the immunosuppressive TGF-ß, providing a poor patient prognosis across most tumors, including GBM (41). Additionally, CAFs can recruit myeloid-derived cells and induce immunosuppressive functions of TAMs (42).

Regarding collagen, while the normal brain tissue expresses minimal amounts of collagen, gliomas overexpress it –including GBM (43). GBM cells synthesize collagen III, IV, V, VI, XVI, and XVII (44–46), and it has been implicated in GBM tumorigenesis. For example, inhibiting Collagen XVI in a human GBM cell line decreased cell invasiveness, and Collagen V and XVII have shown to modulate malignancy through ECM remodeling (47, 48). Furthermore, the ECM crosslinking enzyme lysyl oxidase regulated tumor angiogenesis through the disruption of collagen expression of GBM cells *in vivo* (49). In GBM, the collagen chaperone protein HSP47 overexpression has been shown to promote tumor formation through the TGF-ß mediated induction of ECM-related genes (50). While the proportion of collagen in GBM matches that of other cancers such as lung and breast, it differs in its decreased organization and more thin fibrillar collagen bundles (51). The characteristics of collagen in GBM seem to play an important role in tumorigenesis and immunosuppression, as GBM patients that had a more organized collagen structure showed higher survival and less tumor invasiveness than those with lesser organized collagen (52).

### Hyaluronic acid

Hyaluronic acid affects immune cells differently depending on its polymer size as it binds to varying membrane receptors. For example, macrophages bind HA through CD44 and the receptor for HA-mediated motility (RHAMM) (53, 54). In a three-dimensional matrix containing HA, monocytes were polarized to an M2 immunosuppressive phenotype and showed an increased expression of CD44 and RHAMM (55). Intermediate weight HA, present in tumor culture supernatants from multiple cancer cell lines, including glioma, induced an early activation of macrophages followed by the formation of immunosuppressive M2-like cells are refractory to activating cytokines. Meanwhile, LMWHA and HMWHA had little to no effect (56). This finding was corroborated by Zhang et al., who confirmed this relationship with monocytes from the peripheral blood of patients with solid tumors (57). In mice models, LMW-HA was shown to induce the expression of inflammatory genes on macrophages and act as an adjuvant by enhancing T cell responses through toll-like receptor 2, the same receptor inhibited by HMW-HA (58).

In GBM, HA is abundant and heterogeneously distributed. Infiltrating T cells and CD68+ macrophages exist in areas of low HA content, and this was leveraged to increase immunotherapy efficacy through the introduction of a hyaluronidase (59). In this study, a statistically significant increase in mean survival time was observed when hyaluronidase was added to an oncolytic adenovirus therapy against GBM. The addition of hyaluronidase increased the lymphocytic infiltrates and the number of M1-polarized TAMs in the TME. However, this was accompanied by an increased expression of PD-L1. After the addition of anti-PD1 to the hyaluronidase-including oncolytic virus, the median survival time was double that of the non-hyaluronidase-containing oncolytic virus with anti-PD1. This is a clear example of how the TME component HA can be leveraged to increase the efficacy of cancer therapies.

### Fibronectin

Fibronectin is an extracellular matrix protein expressed in blood vessels in normal brain tissue and overexpressed in GBM cells (60). It contains a domain that is almost exclusively expressed in newly formed blood vessels in solid tumors and thus has been identified as a potential for targeted immunotherapies (61). Furthermore, fibronectin-1 can be used to predict muscle-invasive bladder cancer patients’ response to immune checkpoint inhibitors (62).

In the lung tumor stroma, researchers found that the lymphocytes were preferentially located in regions of loose collagen and fibronectin networks (63). Additionally, the lymphocytes migrated along fibronectin fibers parallel to the vessel via a mechanism not mediated by integrin. This study demonstrates that matrix fiber composition and structure can restrict T cells from accessing the tumor cells and even guide migration away from the tumor stroma.

**Figure 1:**
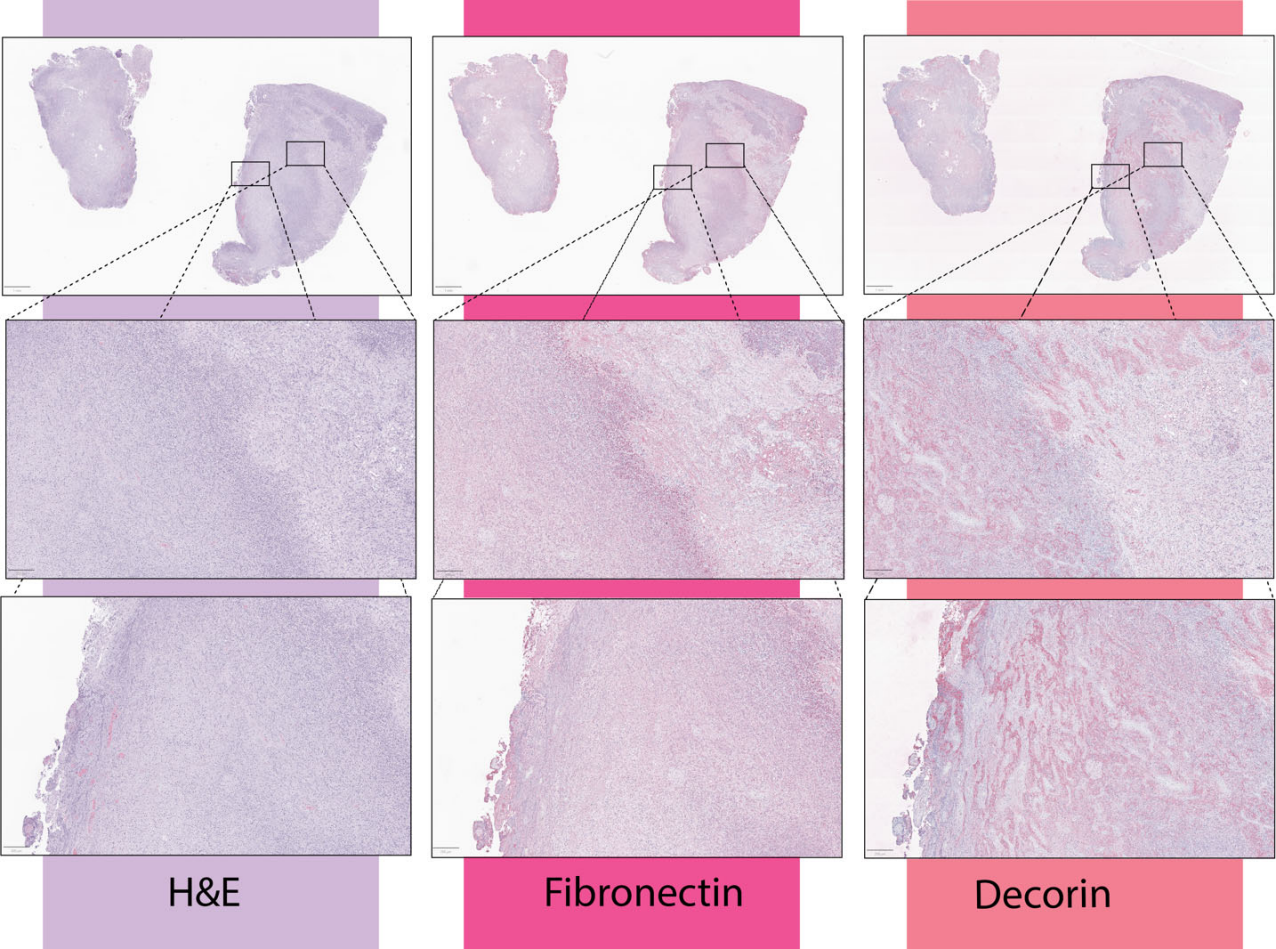
From a cohort of 19 newly-diagnosed GBM patient tumor samples, immunohistochemical stains to fibronectin-1 and decorin were obtained. Per review by a board-certified pathologist, fibronectin was enhanced in areas immediately adjacent to necrosis, while decorin was enhanced within the necrotic core of the tumor. Presented here are three representative samples. Samples were collected by the The Nervous System Tumor Bank at Northwestern University was used to collect all human samples under IRB number STU00202003.

In GBM tumor samples, fibronectin appeared enriched in regions immediately adjacent to areas of necrosis (**Figure 1**). This protein has been shown to promote GBM growth, invasiveness, and vascularization (64, 65). *In vivo*, a fibronectin knockdown glioma cell line on mouse models delayed tumor growth through a lag in regulatory T cell recruitment, and conferred survival benefits (66). Further demonstrating the immunomodulatory capacity of the extracellular matrix.

### Decorin

Decorin is a small proteoglycan that forms part of the extracellular matrix. It has shown anti-tumorigenic properties, disrupting TGF-ß mediated pathways and activating inflammatory responses through TLR2/4 (67). *In vitro*, decorin suppressed TGF-ß signaling in glioma cells (68). Inducing decorin expression in oncolytic adenovirus induced cytotoxicity of tumor cells (36, 69). An immunocompetent orthotopic xenograft breast cancer mouse model induced an immune response and decreased anti-inflammatory responses (70). Contrastingly, when decorin was loaded to oncolytic adenoviruses on mesenchymal stem cells, the inhibition of anti-inflammatory cytokines was abolished. As such, it seems that decorin’s role in inflammation depends on the cellular interactions it establishes (71).

In GBM, decorin has been shown to decrease matrix stiffness, enhance immunogenicity, induce autophagy of tumor cells, decrease VEGf expression, and mediate microglia infiltration (72–75). Furthermore, tumors that express decorin have increased infiltration of activated T cells (76). In GBM tumor samples, decorin expression was greatly increased within the necrotic core (**Figure 1**). While decorin overexpression in GBM has been historically a target for virotherapy (77–79) the role these therapies have on anti-tumor immunity in GBM is unknown.

## Conclusion

**Figure 2:**
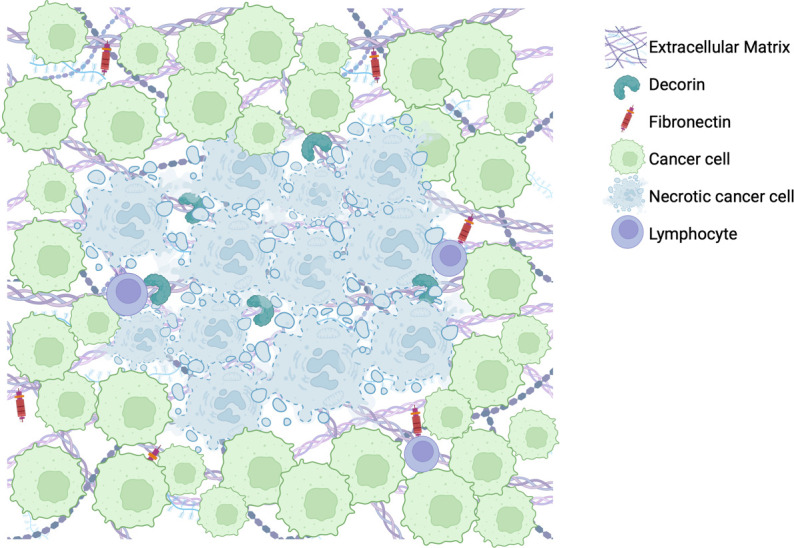
Illustration of representative findings of immunohistochemical stains for fibronectin-1 and decorin within a cohort of 19 newly diagnosed GBM tumor patient samples.

GBM has provided the field with significant challenges as its immunosuppressive features, aberrant tumor stroma, and spatiotemporal heterogeneity impede effective anti-tumor therapeutics. The ECM is an important immunomodulatory component of the TME that can be harnessed to increase the efficacy of immunotherapies. ECM targets of interest that have shown promise in other tumors or have shown to play a role in GBM include hyaluronic acid, fibronectin, decorin, and collagen. Investigating the localization of fibronectin-1 expression in GBM, showed a denser aggregation of it adjacent to necrotic areas, while decorin expression was highest within the necrotic core (**Figure 2**). These samples are exemplary of the heterogeneity observed within the tumor that immunotherapies may target, particularly as a mechanism of increasing lymphocytic infiltration in areas currently untouched by them. Understanding the intra-tumoral variation of these various components can clue us in to ways of harnessing the ECM as a powerful adjunctive tool against GBM.

## Data availability statement

The raw data supporting the conclusions of this article will be made available by the authors, without undue reservation.

## Ethics statement

The studies involving humans were approved by Northwestern University Institutional Review Board. The studies were conducted in accordance with the local legislation and institutional requirements. The human samples used in this study were acquired from The Nervous System Tumor Bank at Northwestern University was used to collect all human samples. Written informed consent for participation was not required from the participants or the participants’ legal guardians/next of kin in accordance with the national legislation and institutional requirements.

## Author contributions

JC: Conceptualization, Data curation, Formal Analysis, Investigation, Methodology, Project administration, Software, Validation, Visualization, Writing – original draft. LB: Conceptualization, Data curation, Formal Analysis, Investigation, Methodology, Software, Validation, Visualization, Writing – original draft. JA: Validation, Writing – review & editing. JM: Conceptualization, Data curation, Formal Analysis, Investigation, Methodology, Resources, Software, Supervision, Visualization, Writing – review & editing. CL-C: Conceptualization, Data curation, Formal Analysis, Funding acquisition, Investigation, Methodology, Project administration, Supervision, Validation, Writing – original draft, Writing – review & editing.
